# Care for Alzheimer's Disease

**DOI:** 10.1155/2013/516852

**Published:** 2013-04-18

**Authors:** Hiroyuki Umegaki, Hajime Takechi, Hiroko H. Dodge

**Affiliations:** ^1^Department of Geriatrics, Nagoya University Graduate School of Medicine, 65 Tsuruma-cho, Showa-ku, Aichi, Nagoya 466-8550, Japan; ^2^Department of Geriatric Medicine, Graduate School of Medicine, Kyoto University, Sakyo-ku, Kyoto 606-8507, Japan; ^3^Department of Neurology, Oregon Health & Sciences University, Portland, Oregon 97239, USA; ^4^Michigan Alzheimer's Disease Center, Ann Arbor, Michigan, USA

Cognitive impairment due to dementia deprives those afflicted with the disease of their autonomy and ability to take care of themselves, making them dependent on care provided by formal and informal resources. Moreover, as the disease progresses, behavioral and psychological symptoms of dementia (BPSD) may occur. BPSD are often troublesome to both caregivers and the patients themselves. The care burden is often heavy and disrupts the lives of family members surrounding the patient.

Early screening and identification of cognitive decline will help those with the disease and family members prepare for better care and may reduce the patients' BPSD. Effective screening for dementia, which can be administered at ambulatory care facilities, is warranted. Vascular risk factors, especially type 2 diabetes mellitus, have been found to increase the risk of developing Alzheimer's disease (AD). The prevalence of type 2 diabetes mellitus is increasing in Japan. In this special issue, T. Matsuzawa et al. describe an index for screening for mild or moderate AD cases among the elderly with type 2 diabetes mellitus. The index includes self-reported answers to a questionnaire regarding subjective memory complaints and daily functioning and information on vascular risk factors obtained from clinical charts. The index had satisfactory discriminatory ability to identify those with AD among patients with diabetes. This may contribute to an effective screening for AD among those with diabetes, that is, known high-risk populations. 

Current treatments for AD are limited to alleviating symptoms, but not reversing the pathological progress. Pharmacological treatments for BPSD, which are critical for the well-being of both patients and caregivers, are also limited in their effectiveness. The study by H. Fukui et al. examines changes in sex hormones associated with the pathogenesis of AD before and after music therapy among patients with AD. They show that music therapy modified the secretion of sex hormones, and the results also suggest that problematic behavior may be reduced this way. The therapy has the potential to become a safe alternative treatment that is as effective as hormone replacement, but with fewer side effects. The underlying biological mechanism of effects shown in this study is convincing and a welcome addition to the field. 

The number of those suffering from dementia is increasing worldwide. Effective caregiving strategies and targeted care management programs are urgently needed to enhance the well-being of those with AD and their caregivers. T. Passos et al. examined the met and unmet needs of the elderly with mental health problems and their care in Portugal. The main unmet needs identified were daytime activities, social benefits, company, psychological distress, and incontinence. Some of these unmet needs may be universal across countries worldwide, but others may differ depending on specific health care systems and cultures. A comparison with similar surveys in other countries may help identify common unmet needs for patients and caregivers. M. M. Pöysti et al. compare the characteristics and burdens of male and female spousal caregivers of patients with dementia in Finland. This is an important issue because the proportion of male caregivers is rapidly increasing in many countries. In the study, male caregivers experienced a lower burden than female caregivers even though female caregivers were responsible for more severe cases. The finding should be confirmed, and the reasons behind it should be explored further, so that factors that may reduce high caregiver burden can be identified. 

Finally, the economic and social costs for caring of the demented are of significant importance as the at-risk population grows rapidly around the globe. Japan has one of the fastest growing elderly populations, and in 2000 it launched a new public long-term care insurance system [1]. S. Shinagawa et al. report that the requisite costs in the Japanese long-term care insurance are different between AD and vascular dementia (VD). 

In this special issue, we highlight several important themes in the care for dementia. We hope that the results presented contribute to developing and providing better care of the demented and increase awareness of the importance of international studies of dementia caregiver issues.

*Hiroyuki Umegaki* *Hiroyuki Umegaki* *Hajime Takechi* *Hajime Takechi* *Hiroko H. Dodge* *Hiroko H. Dodge*
